# Gut Microbiota in the Regulation of Intestinal Drug Transporters: Molecular Mechanisms and Pharmacokinetic Implications

**DOI:** 10.3390/ijms262411897

**Published:** 2025-12-10

**Authors:** Patryk Rzeczycki, Oliwia Pęciak, Martyna Plust, Marek Droździk

**Affiliations:** Department of Experimental and Clinical Pharmacology, Pomeranian Medical University, 72 Powstańców Wielkopolskich Avenue, 70-111 Szczecin, Poland; patryk.rzeczycki@pum.edu.pl (P.R.); peciak.oliwia@gmail.com (O.P.); mplust16@gmail.com (M.P.)

**Keywords:** gut microbiota, intestinal drug transporters, intestinal barrier integrity, pharmacokinetics, drug–microbiome interactions

## Abstract

Gut microbiota, through both its species composition and its metabolites, impacts expression and activity of intestinal drug transporters. This phenomenon directly affects absorption process of orally administered drugs and contributes to the observed inter-individual variability in pharmacotherapeutic responses. This review summarizes mechanistic evidence from in vitro and animal studies and integrates clinical observations in which alterations in gut microbiota are associated with changes in oral drug exposure, consistent with potential regulation of key intestinal drug transporters—such as P-glycoprotein (P-gp, ABCB1), Breast Cancer Resistance Protein (BCRP, ABCG2), MRP2/3 proteins (ABCC2/3), and selected Organic Anion-Transporting Polypeptides (OATPs, e.g., SLCO1A2, SLCO2B1)—by major bacterial metabolites including short-chain fatty acids (SCFAs), secondary bile acids, and tryptophan-derived indoles. The molecular mechanisms involved include activation of nuclear and membrane receptors (PXR, FXR, AhR, TGR5), modulation of transcriptional and stress-response pathways (Nrf2, AP-1) with simultaneous suppression of pro-inflammatory pathways (NF-κB), and post-translational modifications (e.g., direct inhibition of P-gp ATPase activity by *Eggerthella lenta* metabolites). The review also highlights the pharmacokinetic implications of, e.g., tacrolimus, digoxin, and metformin. In conclusion, the significance of “drug–transporter–microbiome” interactions for personalized medicine is discussed. Potential therapeutic interventions are also covered (diet, pre-/probiotics, fecal microbiota transplantation, modulation of PXR/FXR/AhR pathways). Considering the microbiota as a “second genome” enables more accurate prediction of drug exposure, reduction in toxicity, and optimization of dosing for orally administered preparations.

## 1. Introduction

The human gastrointestinal tract harbors a dense and diverse community of microbes (the gut microbiota) that plays a pivotal role in host physiology [1]. Beyond involvement in digestion and immunity modulation, gut bacteria and their metabolites can profoundly influence drug pharmacokinetics. However, clinical pharmacokinetic studies rarely allow direct attribution of altered drug exposure to transporter regulation. Observed differences in bioavailability may instead reflect other microbiota-dependent mechanisms, such as luminal drug metabolism, microbial degradation, changes in intestinal permeability, or modifications in enterohepatic cycling. Therefore, clinical observations should be interpreted cautiously and viewed as consistent with, rather than confirmatory of, transporter-mediated effects. Two major families of membrane transport proteins participate in regulating the intestinal handling of xenobiotics: solute carrier (SLC) influx transporters and ATP-binding cassette (ABC) efflux transporters. SLC transporters mediate the uptake of structurally diverse drugs and endogenous substrates into enterocytes, whereas ABC proteins primarily function as ATP-driven efflux pumps that limit intestinal drug accumulation and shape first-pass pharmacokinetics [2]. In the intestine, key SLC representatives include OATP1A2 and OATP2B1 (SLCO1A2/SLCO2B1), PEPT1 (SLC15A1), OCTs and OATs (SLC22 family), and MATEs (SLC47), while the major ABC transporters involved in drug disposition are ABCB1 (P-gp), ABCG2 (BCRP) and ABCC2 (MRP2). Together, these coordinated influx–efflux systems determine the extent of oral drug absorption and contribute significantly to inter-individual variability in systemic drug exposure. Given that gut microbiota and their metabolites can modulate the expression and activity of both SLC and ABC transporters, understanding their baseline physiological roles is crucial for interpreting the microbiota–drug transport interactions discussed in this review. In particular, emerging evidence indicates that the gut microbiota regulates the expression and activity of intestinal drug transporters, thereby affecting oral drug absorption and disposition [3,4].

**Figure 1:**
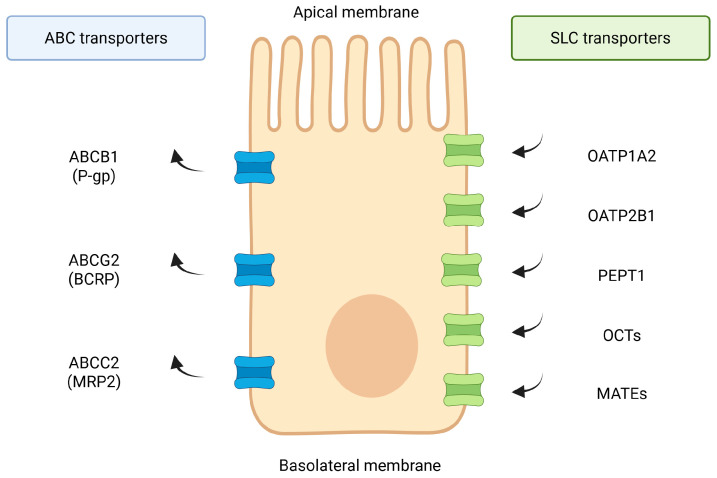
Overview of major intestinal drug transporters. Schematic localization of key apical and basolateral intestinal transporters. ABC efflux pumps (ABCB1/P-gp, ABCG2/BCRP, ABCC-family) mediate ATP-dependent export of xenobiotics toward the lumen, whereas SLC influx systems (OATP1A2, OATP2B1, PEPT1, OCTs/OATs, MATEs) facilitate uptake of clinically relevant drugs. Transporter families and orientations summarized based on established data [1,2,5,6,7,8,9,10,11,12,13,14] and additional reference sources (Zhou et al., 2017 [2]; Zhou & Shu, 2022 [14]). Created in BioRender. Plust, M. (2025) https://BioRender.com/610jab7.

An overview of the major intestinal drug transporters, including their localization and functional orientation, is presented in Figure 1.

Intestinal transport proteins such as P-glycoprotein (P-gp, *ABCB1*), breast cancer resistance protein (BCRP, *ABCG2*), organic anion-transporting polypeptides (OATPs), and multidrug resistance-associated proteins (MRPs) are expressed in the intestinal epithelium/enterocytes, where they govern uptake and efflux of xenobiotics [2,5,6,15]. Alterations in the function of these transporters can lead to significant changes in drug bioavailability and inter-individual variability in drug responses [16]. Notably, conventional pharmacogenomic factors alone often fail to explain observed variability in drug disposition, pointing to the microbiome as a “second genome” influencing drug transport and metabolism. This review provides an in-depth analysis of how specific gut microbial species and their metabolites regulate key intestinal drug transporters at the molecular level [17,18]. We discuss cellular mechanisms involved, including receptor-activated transcriptional pathways and post-translational modifications, and highlight examples of drug–microbiota–transporter interactions that impact pharmacokinetics. We further integrate findings from in vitro studies, animal models, and clinical observations and consider implications for personalized medicine and drug development.

## 2. Gut Microbiota and Intestinal Drug Transporters

**Figure 2:**
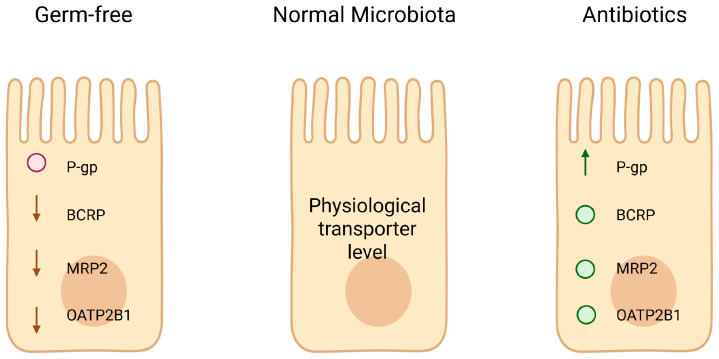
Baseline alterations in intestinal drug transporters under germ-free and microbiota-depleted conditions. Germ-free mice exhibit reduced apical expression of key drug transporters, including ABCB1/P-gp, ABCG2/BCRP, MRP2, and OATP2B1, whereas conventional microbiota restore physiological transporter levels. Antibiotic treatment produces the opposite pattern for some transporters, with increased P-gp expression and variable effects on BCRP, MRP2, and OATP2B1. These baseline differences highlight the essential role of the gut microbiota in maintaining intestinal transporter homeostasis [20,21]. Created in BioRender. Plust, M. (2025) https://BioRender.com/610jab7.

The level of expression of intestinal drug transporters is dynamic and can be shaped by gut microbial signals [19]. Germ-free animals or antibiotic-treated models often exhibit altered transporter levels compared to animals with a normal microbiota, underscoring a microbiota-dependent regulation [20,21]. Figure 2 summarizes baseline alterations in intestinal transporter expression observed in germ-free and microbiota-depleted models.

For example, colonization of germ-free mice restores normal intestinal transporter expression patterns, while broad-spectrum antibiotic depletion of the microbiota can dysregulate transporter genes [22]. Specific commensal bacteria have been linked to the modulation of transporters. Notably, Foley et al. identified a “functional core” microbiome (enriched in *Clostridia* and *Bacilli* classes) in mice that was necessary and sufficient for inducing P-glycoprotein expression in the intestinal epithelium [1,23]. Metagenomic analysis of this core community revealed an enhanced capacity for producing short-chain fatty acids (SCFAs) and secondary bile acids, metabolites which positively correlated with P-gp levels. In contrast, dysbiosis characterized by the loss of such beneficial microbes can lead to transporter downregulation [24,25,26].

In ulcerative colitis (UC), a condition marked by reduced *Firmicutes* (including butyrate-producing *Clostridia*) and lower luminal SCFA/bile acid levels, intestinal P-gp expression is significantly diminished [27,28,29]. This reduction in P-gp in UC patients was found to coincide with a loss of microbiota-derived metabolites capable of inducing P-gp, suggesting that a healthy microbiome tonically sustains certain transporter levels [1,30].

Together, these findings establish that the gut microbiota exerts a bidirectional control over intestinal transporters: commensal-derived signals can upregulate transporter expression contributing to mucosal barrier function, whereas microbial imbalances or pathogenic signals may downregulate transporters and compromise drug handling [31,32,33]. The ATP-binding cassette (ABC) family—notably P-gp (ABCB1), BCRP (ABCG2), and MRP2/3 (ABCC2/3) as well as uptake transporters such as OATP2B1 (SLCO2B1) and OATP1A2 on the apical enterocyte membrane are the key intestinal drug transporters regulated by microbiota [1,2,5,6,7,8,9,10,11,12,13,16,17]. From a structural and functional standpoint, intestinal transporters comprise both ATP-dependent efflux pumps of the ABC family and numerous influx systems belonging to the solute carrier (SLC) superfamily. Key SLC drug transporters expressed in enterocytes include PEPT1 (SLC15A1), OATP1A2 and OATP2B1 (SLCO1A2/2B1), as well as members of the SLC22 family such as OCT1/3 and OATs. In addition, transporters involved in luminal cation handling, including PMAT (SLC29A4) and MATE1/2-K (SLC47A1/2), contribute to intestinal uptake and retention of several therapeutics. These SLC transporters play essential roles in governing the absorption of peptide-like drugs, statins, antihistamines, metformin, and other cationic or amphipathic agents [2,14]. This transporter repertoire works in concert to determine the fraction of an orally ingested drug that reaches systemic circulation.

The gut microbiota can impact both transcriptional regulation of the transporter genes and post-translational modulation of transporter proteins, as discussed below. It is important to note that changes in transporter activity due to microbial factors may either increase drug exposures (by reducing efflux or enhancing uptake) or decrease drug levels (by inducing efflux or reducing uptake), depending on the specific context [14,34,35].

## 3. Microbial Metabolites and Transporter Regulation: SCFAs, Bile Acids, and Tryptophan Derivatives

**Figure 3:**
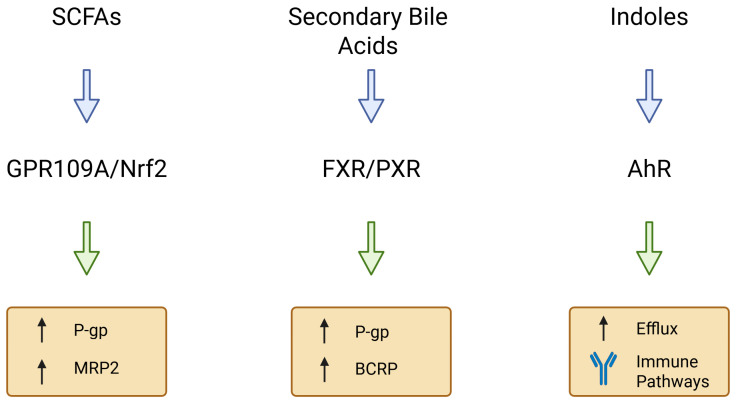
Microbial metabolites and signaling pathways regulating intestinal drug transporters. Short-chain fatty acids (SCFAs) regulate P-gp and MRP2 primarily via GPR109A and Nrf2-dependent signaling [40,41]. Secondary bile acids activate FXR and PXR, modulating ABCB1/P-gp, ABCG2/BCRP and OATP transporters [42,43]. Indole derivatives signal through AhR, influencing efflux transporter expression and immune-associated regulatory pathways [44]. Together, these metabolite groups represent major microbiota-derived modulators of intestinal transporter homeostasis. Created in BioRender. Plust, M. (2025) https://BioRender.com/610jab7.

Gut microbes produce metabolites that can act as signaling molecules to the host [36]. Short-chain fatty acids, secondary bile acids, and tryptophan catabolites have emerged as key mediators of microbiota–transporter interactions [37,38]. Those metabolites engage host receptors and signaling pathways (e.g., G-protein coupled receptors and nuclear receptors) to modulate the expression of drug transporter genes in the intestinal epithelium [39]. Figure 3 illustrates the major classes of microbiota-derived metabolites and their associated signaling pathways regulating intestinal drug transporters.

### 3.1. Short-Chain Fatty Acids Upregulating Drug Transporters

SCFAs (acetate, propionate, and notably butyrate) are produced by fermentation of dietary fibers by anaerobic bacteria (primarily Firmicutes like *Faecalibacterium prausnitzii* and *Roseburia* spp.) [45].

Butyrate has long been recognized as a key signaling metabolite in the gut; it serves as the preferred energy source for colonocytes and modulates gene expression via epigenetic and receptor-mediated mechanisms [46,47]. Butyrate can also robustly induce P-glycoprotein expression and activity in intestinal cells. Mechanistically, butyrate is an inhibitor of histone deacetylases (HDAC), leading to hyperacetylation of histones and altered transcription of target gene [48], including promoter regions of *ABCB1* gene (encoding P-gp), thereby enhancing transcription. In addition, butyrate and other SCFAs activate certain G-protein coupled receptors on intestinal epithelial cells (such as FFAR2/GPR43, FFAR3/GPR41, and GPR109A) [1,49,50,51]. GPCR activation triggers intracellular signaling cascades (e.g., via PKC or MAPKs) that can converge on transcription factors regulating transporter genes. Butyrate has also been shown to activate Nrf2 (nuclear factor erythroid 2-related factor 2), a redox-sensitive transcription factor, which activation leads to increased expression of various cytoprotective genes, including certain transporters [52,53]. In colon carcinoma cell models, pharmacologic activation of Nrf2 upregulated P-gp expression, consistent with this pathway’s involvement [54,55].

Notably, the upregulation of P-gp by butyrate has been demonstrated in vitro and in vivo in the context of not only xenobiotic handling but also in mitigating intestinal inflammation (by promoting the efflux of anti-inflammatory endogenous substrates) [23,56,57]. For example, butyrate-producing commensals are depleted in ulcerative colitis, and this correlates with reduced colonic P-gp and a pro-inflammatory state. Replenishing butyrate (through fiber diet or probiotics) in animal models restores P-gp levels and improves colitis, underscoring SCFAs’ regulatory role [58]. Furthermore, probiotics such as *Lactobacillus acidophilus* that increase luminal butyrate or otherwise stimulate epithelial cells can enhance P-gp expression. Studies have shown that *Lactobacilli* treatment of intestinal epithelial monolayers leads to higher P-gp levels and function via activation of AP-1 transcription factors (c-Fos/c-Jun). This AP-1 activation may be downstream of SCFA signaling or other microbe-associated molecular patterns, illustrating how commensal bacteria orchestrate host transporter defenses [59,60,61,62].

Beyond P-gp, SCFAs may also influence other transporters. There is evidence that butyrate can modulate the expression of MRP family transporters and uptake carriers, although data are less extensive than for P-gp. In the kidney, SCFAs have been reported to enhance OAT (organic anion transporter) expression and facilitate toxin excretion [63,64,65]. By analogy, in the gut, SCFA signaling might upregulate efflux pumps like MRP2 (which exports organic anions and conjugated drug metabolites) via Nrf2 or other pathways, since Nrf2 is a known inducer of MRP2 in response to oxidative stress. Indeed, treatment of colitic rats with SCFA-producing fiber (inulin) prevented the downregulation of Mrp2 that otherwise occurs in colitis, suggesting SCFAs help maintain MRP2 expression under inflammatory conditions [66,67,68]. Thus, SCFAs broadly act as beneficial modulators, promoting a transporter expression profile that enhances mucosal barrier function and xenobiotic clearance [44]. SCFAs also regulate specific SLC transporters. In a conventional-vs-germ-free mouse model, colonic expression of the butyrate transporter SLC5A8 and the SCFA receptor GPR109A was markedly reduced under germ-free conditions and restored by microbial colonization, indicating that SCFA-producing taxa directly shape epithelial uptake capacity [40]. Furthermore, human biopsy data demonstrate that individuals with higher fecal SCFA levels exhibit increased colonic expression of the organic cation transporter OCT3 (SLC22A3), suggesting a link between microbial SCFA availability and intestinal uptake of cationic drugs such as metformin [41].

### 3.2. Secondary Bile Acids and Nuclear Receptor Signaling

Bile acids are another class of microbiota-dependent molecules that regulate drug transporter genes. Primary bile acids (e.g., cholic acid, chenodeoxycholic acid) are synthesized from cholesterol in the liver, secreted in bile, and can be reabsorbed in the intestine [69]. The gut microbiota enzymatically converts primary bile acids into secondary bile acids (such as deoxycholic acid, lithocholic acid, and ursodeoxycholic acid) via deconjugation and dihydroxylation reactions [70]. These secondary bile acids not only facilitate lipid absorption but also serve as potent signaling ligands for host nuclear receptors, notably the farnesoid X receptor (FXR) and pregnane X receptor (PXR), as well as membrane G-protein coupled receptors like TGR5. Through these receptors, bile acids can modulate the transcription of numerous genes including those encoding drug transporters [42]. Beyond ABC transporters, bile-acid-dependent FXR and PXR signaling also regulates several intestinal SLC systems, including the apical bile acid transporter ASBT (SLC10A2), the basolateral OSTα/β complex, and selected OATP and OAT family members, integrating microbiota-driven bile acid remodeling with coordinated control of both drug uptake and efflux [43,71].

Certain secondary bile acids, such as lithocholic acid (LCA) and deoxycholic acid (DCA), are known agonists of PXR. PXR is a xenobiotic-sensing nuclear receptor highly expressed in the intestine (and liver) that, upon activation, forms a heterodimer with RXR and binds to response elements in the promoter regions of target genes [1,72]. PXR activation typically induces the expression of genes involved in drug metabolism and transport, including CYP3A4 (a major drug-metabolizing enzyme) and multiple drug transporters. Notably, activation of PXR has been shown to increase the transcription of P-glycoprotein (*MDR1/ABCB1*) and BCRP (*ABCG2*) in intestinal models [1,73]. Foley et al. demonstrated that LCA and DCA at physiologically relevant concentrations could induce P-gp expression in colonic epithelial cells, especially when combined with butyrate. This synergistic effect aligns with a model where butyrate and secondary bile acids activate parallel pathways (HDAC inhibition/GPCRs and PXR, respectively) that converge on boosting MDR1 gene transcription [1,23]. Thus, a microbiota rich in bile acid-metabolizing bacteria (e.g., certain *Clostridium* clusters that carry bile salt hydrolases and 7α-dehydroxylation enzymes) is able to generate higher levels of secondary bile acids, which in turn activate PXR in the intestine and upregulate protective transporters like P-gp and MRP2 [74,75].

FXR is another bile acid-activated nuclear receptor expressed in the ileum and liver. While FXR primarily governs bile acid homeostasis (controlling synthesis and enterohepatic cycling), it can indirectly influence drug transporter expression. In the intestine, FXR activation (for instance by chenodeoxycholate or microbial metabolites like certain secondary bile acids) induces the production of fibroblast growth factor 19 (FGF19), which signals to the liver to reduce bile acid synthesis [76,77]. Additionally, intestinal FXR activation upregulates genes involved in bile acid export such as organic solute transporter α and β (OSTα/β, the basolateral bile acid efflux transporter) and may downregulate the apical bile acid uptake transporter sodium-dependent bile acid transporter (ASBT), to protect against bile acid overload [78]. Regarding drug transporters, FXR agonism has been reported to increase MRP2 expression in liver, aiding the biliary excretion of bile acids. In enterocytes, FXR’s effect on classical drug transporters is less direct, but by altering the bile acid pool and local inflammation, FXR can modulate transporter regulation [79,80]. For example, FXR activation tends to have anti-inflammatory effects in the gut. Reduced inflammation can relieve NF-κB-mediated suppression of certain transporters (NF-κB, a pro-inflammatory transcription factor, often represses transporter gene expression during inflammation). Thus, via maintaining anti-inflammatory tone, FXR-active bile acids from the microbiota might indirectly preserve higher P-gp/BCRP levels [81,82]. Moreover, FXR shares some target gene overlap with PXR and CAR; studies have indicated FXR ligands can cross-activate PXR target genes to a degree. Overall, the microbiota–bile acid–FXR/PXR axis is a crucial pathway wherein microbial metabolism of bile acids leads to activation of host receptors that transcriptionally induce drug transporters like P-gp and MRPs [83,84].

Beyond P-gp, MRP2 (*ABCC2*), an apical efflux pump exporting organic anions and phase II conjugates [85] in the intestine, can also be upregulated by bile-acid-activated receptors. Its expression in enterocytes could be enhanced by PXR (which is known to induce hepatic MRP2) and by FXR (to facilitate extrusion of glucuronidated bile acids and bilirubin) [71].

In a healthy microbiome setting, ample secondary bile acids and luminal butyrate likely maintain not only P-gp but also an array of efflux transporters that function coordinately to protect the mucosa from both toxins and inflammatory mediators [22,86]. Indeed, an observed phenomenon in ulcerative colitis is an imbalance in the P-gp/MRP2 axis: UC patients typically show reduced P-gp expression but paradoxically increased MRP2 expression in inflamed colon [87]. One hypothesis suggests that dysbiosis in UC (low SCFA, low secondary bile acids) fails to sustain P-gp (which has anti-inflammatory roles), while inflammatory signals (or residual FXR activation by accumulated primary bile acids) selectively induce MRP2 as a stress response [88].

### 3.3. Tryptophan Metabolites and Aryl Hydrocarbon Receptor (AhR) Activation

Dietary tryptophan is another substrate for gut microbial metabolism, leading to various indole and indole-derivative compounds [89,90]. The microbial tryptophan metabolites (e.g., indole, indole-3-acetic acid, indole-3-propionic acid, tryptamine, and others) are important modulators of gut epithelial biology and immune homeostasis [91,92]. A key mechanism by which they signal to the host is through activation of the aryl hydrocarbon receptor (AhR), a ligand-activated transcription factor expressed in intestinal epithelial and immune cells. AhR is traditionally known for sensing environmental toxins (like dioxins), but endogenous and microbial indoles are natural AhR ligands in the gut lumen [93,94]. Activation of AhR has been shown to transcriptionally upregulate the gene encoding BCRP (*ABCG2*), a major drug efflux transporter [95,96]. Studies in human intestinal cells and other models demonstrate that AhR acts as a direct transcriptional activator of *ABCG2* [97]. For instance, treating cells with known AhR agonists (such as TCDD or indole-3-carbinol) increases BCRP mRNA and protein levels, whereas knocking down AhR reduces baseline BCRP expression [98]. A xenobiotic response element (XRE) has been identified in the *ABCG2* promoter, through which ligand-activated AhR can enhance BCRP transcription [99]. Therefore, gut microbial metabolites that engage AhR can booster the intestinal efflux of substrates via BCRP [100,101]. Indole and certain indole derivatives from commensal bacteria (e.g., *Clostridium sporogenes* produces indole-3-propionic acid, a potent AhR agonist) likely contribute to baseline BCRP expression in the gut [102]. This is supported by observations that colonization with AhR ligand-producing microbiota increases epithelial expression of detoxifying enzymes and transporters, reinforcing the intestinal barrier [103].

In addition to BCRP, AhR activation may influence other transporters and metabolizing enzymes that contain XREs in their regulatory regions (such as certain phase I/II enzymes and possibly *ABCB1*). However, BCRP is the clearest example of an efflux pump directly upregulated by AhR [104,105]. By effluxing dietary carcinogens and microbial metabolites, BCRP induction via AhR is considered a key cytoprotective response [106,107].

It should be noted that tryptophan metabolites also modulate immune pathways (e.g., IL-22 production via AhR in innate lymphoid cells) which can secondarily affect transporter expression by altering the inflammatory milieu [108,109].

In the context of drug transport, its activation by microbiota-derived indoles enhances the gut’s capability to pump out potentially harmful compounds via transporters like BCRP and possibly P-gp. Some probiotics (e.g., certain *Lactobacillus strains*) produce indole-3-aldehyde, activating AhR and promoting mucosal barrier integrity, this could translate to maintained or elevated transporter levels as part of the barrier function [110,111]. Overall, tryptophan metabolite signaling through AhR constitutes a crucial link between microbiota metabolism and the upregulation of intestinal transporters involved in xenobiotic defense [112].

### 3.4. Other Microbial Metabolites and Factors

While SCFAs, bile acids, and indoles are among the most studied, other microbial products can potentially impact transporter functions as well:

- Phenolic Metabolites: Gut bacteria metabolize polyphenols and aromatic compounds into phenolic acids that may activate receptors like PXR or Nrf2 [40]. For example, urolithins (derived from polyphenols by gut microbes) have been noted to induce phase II enzymes and could affect transporters via Nrf2 activation, though direct evidence on transporter genes is still emerging [113].
- Trimethylamine N-oxide (TMAO): Produced from dietary choline/carnitine by a two-step microbial-host process, TMAO has been implicated in modulating bile acid and cholesterol transport. Some studies suggest TMAO may downregulate hepatic transporters (like OATP and NTCP) via FXR signaling. In the intestine, a high-TMAO environment (as in some dysbiosis) could conceivably alter FXR and hence transporter expression, although details remain to be clarified [114].
- Microbial Enzymes and Postbiotics: Microbes secrete enzymes or peptide signaling molecules that interact with host cells. Probiotics can release soluble proteins that activate MAPK pathways in epithelial cells, potentially influencing transporter gene expression similar to how *Lactobacillus* activated AP-1 to increase P-gp. Additionally, microbial fermentation gases (H_2_S, methane) or small molecules (like spermine, polyamines) might impact cellular signaling pathways [115,116,117,118], that modulate transporter post-translational modification (e.g., phosphorylation status affecting transporter activity) [119].
- Pathogen-associated Molecular Patterns (PAMPs): Components of microbial cells, such as lipopolysaccharide (LPS) from Gram-negative bacteria, can also affect transporter function. LPS triggers Toll-like receptor 4 (TLR4) and downstream NF-κB inflammatory signaling in the gut [120,121]. Acute exposure to LPS or a pro-inflammatory state has been shown to downregulate P-glycoprotein expression and inhibit its function in various tissues [122]. For example, LPS administration in rodents impaired P-gp-mediated efflux at blood–tissue barriers. In the intestine, inflammation due to pathogenic bacteria or microbial imbalance could similarly suppress P-gp and other transporter levels as part of the broader NF-κB driven response [122,123]. Infections like *Citrobacter rodentium* in mice reduce colonic P-gp expression, an effect linked to increased epithelial permeability and inflammation [124]. Such findings highlight that not all microbial signals induce transporters—some, particularly from pathogens or dysbiosis, can inhibit transporter expression and activity, diminishing the gut’s drug efflux capability [125,126]. The summary is provided in Table 1 below.

## 4. Molecular Pathways of Microbiota-Driven Transporter Regulation

It should be emphasized that most of the mechanistic pathways described in this section are supported predominantly by preclinical in vitro and animal studies. Direct confirmation of these mechanisms in the human intestinal epithelium remains limited, and therefore they should be interpreted as biologically plausible rather than definitively established. Understanding which host molecular pathways are involved provides mechanistic insight into how microbes influence transporter expression. Several key pathways have emerged: nuclear receptors (e.g., PXR, FXR, CAR, VDR), transcription factors (e.g., Nrf2, NF-κB, AP-1), and kinase signaling cascades (affecting post-translational modifications of transporters). These pathways can be activated or inhibited by microbial metabolites, leading to up- or down-regulation of transporter proteins [128]. Although ABC efflux pumps such as P-gp, BCRP, and MRPs have been most extensively characterized, these microbiota-responsive pathways also regulate clinically important SLC transporters. Mechanistic studies indicate that microbial metabolites influence the transcription and membrane abundance of various SLCO and SLC22 carriers, suggesting that the microbiota orchestrates a coordinated modulation of both uptake and efflux systems (Zhou i in., 2017 [2]; Zhou i Shu, 2022 [14]).

**Figure 4:**
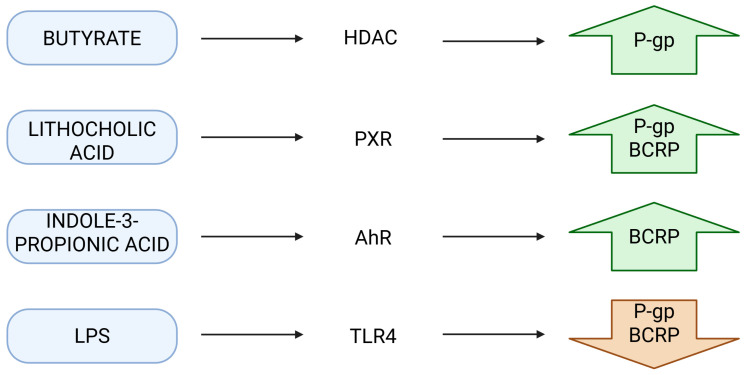
Microbial metabolite–receptor interactions regulating intestinal efflux transporters. Butyrate activates HDAC-dependent signaling, inducing P-glycoprotein (P-gp). Lithocholic acid activates PXR, upregulating P-gp and BCRP. Indole-3-propionic acid signals through AhR to increase BCRP expression. LPS activates TLR4, modulating inflammatory signaling that influences P-gp and BCRP. Together, these pathways represent key nodes linking microbiota-derived metabolites to transporter regulation. Created in BioRender. Plust, M. (2025) https://BioRender.com/zl2fzz8.

As shown in Figure 4, representative microbial metabolites—including butyrate, lithocholic acid, indole-3-propionic acid and LPS—activate distinct host receptors (HDAC, PXR, AhR, TLR4), leading to differential regulation of intestinal efflux transporters such as P-gp and BCRP.

### 4.1. Nuclear Receptors as Xenobiotic and Metabolite Sensors

Nuclear receptors (NRs) are ligand-activated transcription factors that play central roles in xenobiotic sensing and metabolic gene regulation. In the context of gut microbiota and drug transporters, the most relevant NRs include:

- Pregnane X Receptor (PXR): PXR is activated by various microbial metabolites (secondary bile acids like LCA, certain dietary compounds possibly modified by microbes, etc.). When activated in intestinal enterocytes, PXR binds to response elements on genes to induce a suite of xenobiotic-handling proteins—like *ABCB1* (P-gp), *ABCC2* (MRP2), *ABCG2* (BCRP), and CYP3A4 [3,43,129,130,131]. In addition to ABC transporters, PXR activation has been shown to regulate multiple SLC genes, including members of the SLCO (OATP) and SLC22 (OAT/OCT) families, indicating that microbiota-derived PXR ligands may reshape both intestinal drug uptake and efflux (Zhou i in., 2017 [2,58]; Zhou i Shu, 2022 [14]). PXR thus serves as a crucial mediator by which microbiota can enhance efflux transporter expression to handle increased luminal loads of foreign chemicals. Interestingly, gut microbes themselves can modulate PXR signaling not only by providing ligands but also by influencing PXR expression levels [132].
- Constitutive Androstane Receptor (CAR): CAR is another xenobiotic-sensing NR which often overlaps with PXR in target genes but is usually active basally and further induced by certain ligands (e.g., phenobarbital-type inducers) [133]. They pinpointed CAR as a likely transcription factor mediating microbiota-induced changes in *Abcb1* (P-gp) expression [134]. It appears that some microbiota metabolites may repress CAR activity under normal conditions, and removing them (with antibiotics) triggers CAR, which then boosts P-gp expression. CAR’s exact endogenous ligands from microbiota are not well-characterized; indirect modulation via altered bile acid pools or cytokine signaling is possible [135]. CAR activation is generally associated with induction of certain efflux pumps and phase II enzymes, so it aligns with an increase in transporter expression [136].
- Farnesoid X Receptor (FXR): FXR, activated by bile acids, influences primarily bile acid transporters. Microbiota-controlled bile acid profiles will determine FXR activation. Intestinal FXR activation tends to maintain barrier integrity and can have varying effects on drug transporters [137]. Some studies in FXR-knockout mice show alterations in P-gp and BCRP levels during cholestatic conditions, implying FXR may contribute to their regulation. Notably, FXR agonists (e.g., obeticholic acid) given to mice shift the gut microbiota and also change expression of some ABC transporters, though disentangling cause/effect is complex [138].
- Vitamin D Receptor (VDR): VDR can be activated by lithocholic acid (a secondary bile acid) which is a VDR ligand. Activation of VDR has been shown to induce P-gp expression in the gut as well, since vitamin D/VDR signaling upregulates ABCB1 in various tissues. Microbiota that produce LCA could activate VDR locally, potentially contributing to P-gp regulation [139,140].

### 4.2. Transcription Factors and Signaling Pathways

- Nrf2 (Nuclear factor E2-related factor 2): Nrf2 is a master regulator of cellular antioxidant and defensive responses. SCFAs like butyrate can activate Nrf2. When Nrf2 translocates to the nucleus, it binds antioxidant response elements (AREs) in gene promoters. Nrf2 drives expression of many phase II metabolism enzymes (UGTs, GSTs) and certain transporters including MRP2 and MRP3. Butyrate-mediated Nrf2 activation has been linked to increased P-gp levels as well. For example, berberine (a plant alkaloid that also modulates gut microbes) was shown to upregulate P-gp via Nrf2-dependent mechanisms in colitic rats [141,142].
- NF-κB (Nuclear Factor kappa B): NF-κB is a key inflammatory transcription factor activated by microbial PAMPs (LPS, flagellin) and cytokines [143]. Activation of NF-κB generally leads to production of pro-inflammatory mediators, but it can also repress certain genes. In inflammatory states of the gut, NF-κB activation correlates with decreased expression of transporters like P-gp and BCRP [144,145]. The mechanism may involve NF-κB interfering with the binding of positive factors (like PXR or constitutive transcription factors) on transporter gene promoters [146]. Additionally, NF-κB induces nitric oxide and oxidative stress that can impair transporter function [147]. Overall, chronic NF-κB activation (e.g., in IBD or infection) is associated with transporter downregulation, contributing to barrier compromise [148].
- AP-1 (Activator Protein 1): AP-1 refers to dimeric transcription factors composed of Fos and Jun proteins that respond to MAPK signaling [149]. The MDR1 (P-gp) gene promoter contains AP-1 binding sites known to enhance its transcription [150,151]. Beneficial microbes can activate AP-1 in epithelial cells, e.g., *L. acidophilus* was shown to induce c-Fos/c-Jun, resulting in higher P-gp expression [152]. On the other hand, certain bacterial toxins or stress may activate JNK pathways leading to AP-1, but in contexts like oxidative stress AP-1 might also be repressive [153].
- PPARs (Peroxisome Proliferator-Activated Receptors): These lipid-sensing NRs (PPARα/δ/γ) can be activated by microbial metabolites (e.g., certain fatty acids) [154]. While not classic drug transporter regulators, PPARγ activation by microbiota (as in fermentation products) has anti-inflammatory effects that indirectly preserve transporter function [155]. Some studies indicate PPARα agonists can increase *ABCB1* expression in the liver; whether similar occurs in gut is under investigation [156,157].
- HIF-1 (Hypoxia Inducible Factor 1): The gut mucosa experiences an altered oxygen gradient in dysbiosis, possibly activating HIF-1. HIF-1 is known to induce certain transporters (like ABCB1 in hypoxic tumors) [158,159]. A fiber-rich, microbiota-driven increase in butyrate actually consumes oxygen in the colon and can stabilize HIF-1, which has been linked to enhanced barrier function [160]. Microbiota-induced HIF-1 helps upregulate P-gp or other transporters as part of adaptation to low oxygen, though direct evidence in vivo is limited [161].

### 4.3. Post-Translational Modifications of Transporters

One remarkable example is the post-translational inhibition of P-glycoprotein by a bacterial metabolite, as described earlier, *Eggerthella lenta* secretes a factor (a family of isoflavonoid molecules) that directly interacts with P-gp and inhibits its ATPase activity [16,162,163,164]. This prevents P-gp from cycling and pumping substrates, effectively reducing efflux function without changing P-gp expression [162,165]. Such inhibition was observed in cell culture and translated to increased drug absorption in mice colonized with *E. lenta* [16]. The inhibitor is structurally similar to plant-derived P-gp inhibitors, highlighting how microbial metabolites can mimic known drug-transporter inhibitors [166,167].

**Figure 5:**
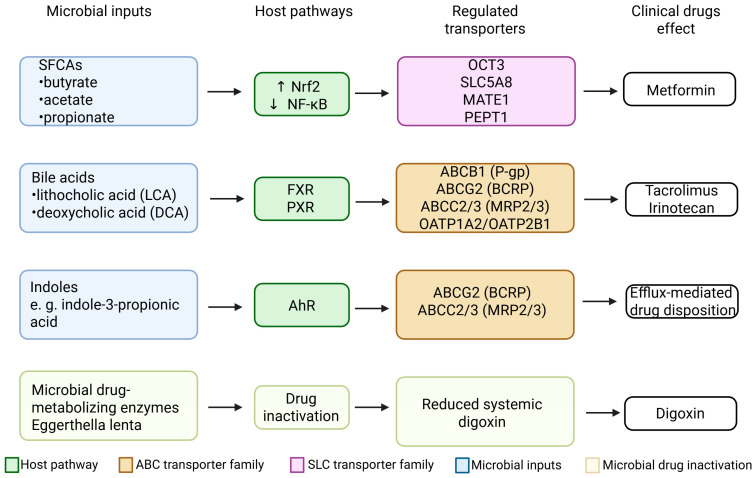
Comprehensive summary of microbial inputs, host signaling pathways, regulated intestinal drug transporters, and affected clinical drugs. Short-chain fatty acids (SCFAs) regulate several SLC transporters, including OCT3, SLC5A8,TE1/2-K, and PEPT1, primarily through activation of Nrf2 and inhibition of NF-κB, influencing the disposition of metformin. Secondary bile acids such as lithocholic acid (LCA) and deoxycholic acid (DCA) activate FXR and PXR, modulating efflux transporters (ABCB1/P-gp, ABCG2/BCRP, ABCC2/3) and uptake transporters (OATP1A2 and OATP2B1), with clinical implications for tacrolimus and irinotecan/SN-38. Indole derivatives activate AhR, upregulating BCRP and MRP2/3 and affecting efflux-mediated drug disposition. *Eggerthella lenta* reduces digoxin to dihydrodigoxigenin, decreasing the systemic fraction of active digoxin independently of host transport mechanisms. Collectively, these pathways illustrate the coordinated influence of gut microbiota on intestinal drug transport and pharmacokinetics. Created in BioRender. Plust, M. (2025) https://BioRender.com/610jab7.

Other PTMs include phosphorylation, glycosylation, ubiquitination of transporters:

- Kinase signaling: Transporter proteins like BCRP and P-gp can be phosphorylated by kinases (e.g., PKA, PKC, JNK) which may alter their localization or activity [6,168]. The gut microbiota modulates host kinase signaling (for example, microbial secondary bile acids can activate PKC or Src kinases) [169]. There is evidence that intestinal BCRP requires phosphorylation by JAK2/3 (Janus kinases) for full activity. In inflammatory states, cytokines activate JAK/STAT pathways which could modify BCRP [170,171]. Microbiota that reduce inflammation might maintain proper JAK-mediated BCRP activation, whereas dysbiosis could lead to aberrant phosphorylation and diminished function [172]. Similarly, P-gp function can be modulated by PKC-mediated phosphorylation; some bacterial toxins activate PKC and might internalize P-gp, reducing efflux at the membrane [173].
- Glycosylation: N-glycosylation of P-gp and BCRP is needed for stability and trafficking to the plasma membrane [174]. Microbiota affect the gut epithelial glycosylation patterns (e.g., via influencing nutrient availability like monosaccharides or modulating endoplasmic reticulum stress). If microbial products interfere with normal glycosylation (for instance, some bacterial infections cause ER stress), transporters could misfold or be targeted for degradation, lowering their surface expression [175].
- Ubiquitin-proteasome degradation: Inflammatory signals triggered by microbes can promote ubiquitin tagging of certain proteins [176,177]. Commensals that activate pathways like AMPK may actually enhance transporter stability by preventing misfolded protein accumulation [178]. It has been noted that butyrate can increase expression of chaperone proteins and stabilize tight junction and transporter proteins in the membrane, though more specific data on transporter PTMs are needed [179].
- Membrane microenvironment: Microbial metabolites can also alter the membrane lipid composition [180]. P-gp activity is known to depend on membrane lipid environment. Secondary bile acids can incorporate into membranes and might modulate how P-gp interacts with the bilayer, potentially changing its conformation and drug affinity [181].
- Although these post-translational mechanisms are biologically plausible and supported by selected in vitro studies, the evidence linking microbiota-derived metabolites directly to transporter PTMs remains fragmentary. Additional work is required to determine the extent to which these modifications occur in vivo and whether they meaningfully influence human drug disposition.
- Figure 5 summarizes the integrated relationships between microbiota-derived metabolites, host signaling pathways, regulated intestinal drug transporters, and representative clinical drugs affected by these interactions.

## 5. Clinical Observations and Human Studies

Direct studies in humans are more limited but growing, given the complexity of human microbiome variability and ethical considerations. Importantly, because intestinal transporter expression or activity is rarely measured directly in human studies, changes in drug levels associated with microbiota alterations cannot be assumed to result solely from transporter modulation. Microbiota-driven effects on luminal metabolism, drug degradation, intestinal permeability, or enterohepatic circulation may equally contribute to these clinical observations. Still, several lines of evidence in clinical or pharmacological studies point to microbiota–transporter interactions:

- Association studies: Analyses of intestinal biopsies have shown correlations between microbiota composition and transporter gene expression. As one example, in a cohort of UC patients versus healthy controls, P-gp expression in colon biopsies was lower in UC and directly correlated with the abundance of butyrate-producing Firmicutes in the patients’ gut microbiota. Those with more *Roseburia/Faecalibacterium* had higher mucosal P-gp, whereas those over-colonized by *Proteobacteria* (like *E. coli*) had lower P-gp. This is consistent with mechanistic data and suggests microbiome profiling might predict transporter expression in individuals [182,183].
- Fecal metabolite associations: Similarly, levels of fecal SCFAs and secondary bile acids in patients have been linked to transporter modulation. Patients on high-fiber diets (with presumably higher SCFAs) showed increased expression of genes like *ABCB1* and *SLC22A3* (OCT3) in colon biopsies in one small study (potentially explaining better drug tolerance) [41]. Conversely, individuals on broad-spectrum antibiotics (which wipe out SCFA producers) had transient reductions in fecal butyrate and coincident drops in P-gp expression (noted in one study examining loperamide response, though more data are needed) [184].
- Drug pharmacokinetics and microbiome status: There are reported instances where changes in a patient’s microbiota (due to antibiotics or illness) altered drug levels in ways consistent with transporter effects:
- Tacrolimus: Clinical anecdotal reports and pilot studies have noted that patients on tacrolimus (an immunosuppressant) experienced elevated drug levels and toxicity after courses of broad-spectrum antibiotics [185]. While some of this is due to loss of bacterial drug metabolism, the study by Degraeve et al. suggests a mechanistic basis: antibiotics increase intestinal P-gp which lowers tacrolimus absorption [17]. In a controlled setting, one study co-administered the P-gp inhibitor zosuquidar to kidney transplant patients and found that normally, P-gp limits tacrolimus absorption, but when gut bacteria are suppressed (as in patients on multiple antibiotics), tacrolimus AUC variance increased markedly until P-gp was blocked. This indicates that microbiota differences could be part of why some patients need higher tacrolimus doses—those with certain microbiomes might have higher baseline P-gp activity [186,187].
- Digoxin: A classic case is digoxin, a cardiac glycoside. About 10% of patients harbor *Eggerthella lenta* strains that metabolize digoxin, reducing its plasma levels. Interestingly, those same strains secrete P-gp inhibitors [188]. A recent trial measured digoxin pharmacokinetics in subjects before and after altering their microbiota (through diet) [189]. In those where *E. lenta* increased, early-phase digoxin blood levels were higher (consistent with P-gp inhibition increasing absorption rate), although total exposure (AUC) did not change much because metabolic degradation counteracted it [190].
- Metformin: Emerging data link metformin response to the microbiome. Metformin’s absorption occurs mainly in the upper intestine via transporters like OCT1 and SERT, and it exerts glucose-lowering effects partly through actions on the gut. Some studies found that microbiome composition (abundance of *Akkermansia* and SCFA producers) correlates with better metformin response [191]. Mechanistically, metformin itself alters bile acid pools and gut microbes, which could upregulate intestinal apical bile acid transporters and limit metformin diarrhea side-effects [192,193].
- Cancer chemotherapeutics: The irinotecan example is notable clinically. Cancer patients on irinotecan sometimes receive antibiotics like neomycin to mitigate diarrhea [194]. Neomycin kills β-glucuronidase-producing bacteria, preventing reactivation of toxic SN-38 in the colon [195]. Additionally, neomycin might modulate transporter expression: small studies hint that neomycin co-treatment leads to higher expression of MRP2 in colon (so that SN-38 glucuronide is effluxed more, reducing exposure of colon mucosa) [196]. Probiotic trials in cancer patients also showed reduced irinotecan diarrhea, presumably by microbiota modulation affecting both metabolism and transport [197]. More broadly, cancer patients often have altered microbiomes due to diet/antibiotics, which may influence oral bioavailability of drugs like tyrosine kinase inhibitors (many of which are P-gp/BCRP substrates) [198].

Direct clinical approaches employing microbiota modifiers are being explored as potential ways to influence drug transport and toxicity [199]. For example, fecal microbiota transplantation (FMT) in UC can restore some butyrate-producing bacteria; one might predict it could also restore colonic P-gp expression, contributing to mucosal healing by exporting inflammatory mediators [200]. Measuring transporter expression pre- and post-FMT could be an interesting clinical research avenue. Probiotics are another form of microbiota modification: administering Lactobacillus or Bifidobacterium strains to patients might increase intestinal P-gp and tighten the barrier—potentially useful in IBD or in reducing drug-induced gut toxicity. However, it could also decrease the absorption of certain oral drugs (a consideration for co-administration timing) [1,17,201,202].

In summary, human data, while still emerging, align with the pattern established in experimental models: a microbiome abundant in SCFA and secondary bile acid producers (often associated with healthy diets) tends to promote a high-expression, high-function state of intestinal efflux transporters, which can reduce the absorption (and toxicity) of certain drugs but also protect the host. On the other hand, a disrupted or inflammatory microbiome can reduce these transporters, possibly increasing drug absorption unpredictably or compromising epithelial defense. This variability underscores the need for personalized approaches in medicine [203,204].

### Case Examples of Drug–Microbiota–Transporter Interactions

Examples where microbiota-mediated changes in transporters have been shown to alter drug pharmacokinetics or response (Table 2).

## 6. Implications for Personalized Medicine and Drug Development

The intricate interplay between gut microbiota and intestinal transporters has several important implications:

### Personalized Medicine and Microbiome Profiling

Inter-individual differences in drug absorption and response (even among those with the same genetic features for drug-metabolizing enzymes and transporters) can be partially explained by differences in gut microbiome composition [205]. As such, analyzing a patient’s microbiome (sometimes termed “pharmacomicrobiomics”) could help personalize drug therapy. For drugs with a narrow therapeutic index or known transporter-mediated variability (e.g., tacrolimus, digoxin, certain anticancer agents), knowing if a patient’s microbiota composition is skewed towards high SCFA producers vs. dysbiosis might guide dose selection [1,16,17,188,206]. For instance, a patient with low butyrate-producing bacteria (perhaps detectable by low fecal butyrate or specific 16S rRNA sequencing) might have lower baseline P-gp expression, thus absorbing more of a P-gp substrate drug, and could be started at a lower dose to avoid toxicity [207]. Conversely, a patient on chronic antibiotics or with microbiome depletion might need higher doses to achieve efficacy due to upregulated efflux transporters. In the future, clinical assays might measure microbial metabolites (like fecal LCA or butyrate levels) as biomarkers for transporter activity in the gut [208].

Interventions to modify the microbiome can be personalized. If a patient is a poor absorber of a life-saving drug due to high efflux, one could consider co-administering a specific probiotic that competes with or reduces the efflux-enhancing bacteria, or even providing an enzyme/pro-drug that the microbiome will convert to a transporter inhibitor in situ (a novel therapeutic strategy hinted by the *E. lenta* digoxin case) [209]. On the other hand, if a patient suffers toxicity from a drug because their microbiome inactivates a transporter (as *E. lenta* does to P-gp), one might use targeted antibiotics or dietary changes to transiently remove that effect (e.g., avoid foods or supplements high in certain flavonoids that feed those bacteria during drug therapy) [188,189,190,210]. The concept of a “microbial pharmacotype” has been proposed—classifying individuals by their microbiome’s capacity to modulate drugs. Incorporating this into precision medicine could improve drug safety and efficacy, especially for drugs where transporter polymorphisms alone do not fully predict outcomes [18,163].

## 7. Drug–Drug–Microbiota Interactions

Classically, drug–drug interactions (DDIs) consider how one drug affects the metabolism or transport of another (often via enzyme or transporter inhibition/induction). We now should extend this model to drug–drug–microbiota interactions. For example, consider a patient taking Drug A that alters the microbiome (like a long-term antibiotic, or a proton pump inhibitor which is known to shift gut flora) and Drug B that is a transporter substrate. Drug A could change microbial metabolite profiles and thus the expression of transporters that handle Drug B [211,212].

A concrete example, a patient on rifampin (a PXR-activating antibiotic) will not only directly induce P-gp via PXR but also markedly alter the gut microbiota composition (rifampin has antibacterial effects) [213]. The microbiome change might reduce SCFA levels (since rifampin can kill commensals), which paradoxically could counteract some induction or alter other transporters [214]. The net effect on Drug B (say a P-gp substrate like dabigatran) could be complex, rifampin’s direct PXR induction increases P-gp, reducing dabigatran levels, but microbiome-mediated effects might further modulate this outcome (perhaps enhancing it if SCFAs drop, since SCFAs were also inducing P-gp normally) [215]. Understanding these three-way interactions is important in polypharmacy.

Another example is metformin and antibiotics: metformin’s efficacy partly depends on microbiota; co-prescription of antibiotics for an infection can cause blood sugar control to fluctuate in diabetic patients on metformin [216]. This is likely due to microbiota disruption affecting both drug metabolism and transporter expression (like OCTs in gut or bile acid transport via FXR) [217]. Thus, clinicians should be aware that adding or removing an antibiotic, or any microbiome-impacting drug (certain psychiatric medications, NSAIDs, etc., also change gut flora), can have knock-on effects on transporter-mediated drug handling [218].

## 8. Pharmacokinetic Modeling and Drug Development

In drug development, physiologically based pharmacokinetic (PBPK) models are used to predict drug absorption and disposition. Traditionally, these models include terms for gut transporter expression and activity that are fixed or vary by genetics. Incorporating the microbiome as a dynamic compartment is the new frontier [219,220]. Some researchers have begun developing PBPK models that include intestinal compartments with microbial drug metabolism activity [221]. This can be expanded to microbial effects on transporters: e.g., models can simulate how varying butyrate concentrations in colon affect P-gp function and thus the fraction of drug absorbed at different gut segments [222]. By plugging in patient-specific microbiome data (obtained via sequencing or metabolomics), one could predict individualized drug AUCs and Cmax with better accuracy than using a one-size transporter expression value [223].

Another implication is in toxicity testing, as animal models for drug toxicity might need to consider microbiota differences. Often, lab animals have different microbiota than humans, which could lead to discrepancies in transporter expression and hence drug exposure [224]. A drug causing intestinal toxicity in rats might do so because those rats lack a certain microbiome protection (like producing mucosal P-gp); gnotobiotic models colonized with human flora or co-housed animals might be useful to bridge this gap [225].

### Microbiota Modifiers Affecting Microbiota–Transporter Pathways

Recognizing these pathways opens the possibility of using microbiota modifiers to influence them for patient benefit:

- Probiotics or Prebiotics: Administering specific strains known to induce transporters (like Lactobacillus for P-gp) could be a strategy to reduce drug absorption when desirable, such as preventing systemic uptake of a toxin or modulating local drug delivery [226]. In inflammatory conditions, probiotic-induced P-gp might help export inflammatory mediators (as P-gp exports endocannabinoids that suppress neutrophils) [227].
- Inhibitors of negative pathways: Blocking the effect of LPS/TLR4 with drugs (e.g., TLR4 antagonists) might prevent the inflammatory downregulation of transporters during infections or sepsis, potentially protecting the barrier and preventing unpredictable drug absorption in critical illness [228].
- Microbiome engineering: Fecal transplants or engineered bacterial consortia could be deployed to manage chronic diseases where transporter regulation is a factor. For example, in IBD, a consortium that produces high butyrate and secondary bile acids might be given to maintain P-gp and promote remission (as low P-gp is implicated in colitis severity) [229]. In metabolic disease, modulating bile-acid metabolizing bacteria might alter FXR signaling and intestinal nutrient transporters to treat obesity/diabetes [230].
- Adjunct therapy in chemotherapy: Given the role of microbial β-glucuronidases and transporters in irinotecan toxicity, combining chemotherapy with microbiome-targeted interventions (antibiotics, enzymatic inhibitors like pPB—a bacterial β-glucuronidase inhibitor, or transporter inducers) is being investigated. Early trials with such adjuncts show promise in reducing side effects without compromising efficacy [231].

In all, the convergence of microbiology and pharmacology heralds a more holistic approach to treatment—where one not only prescribes a drug but also can manipulate the patient’s microbiota or use its signals to optimize therapy.

## 9. Conclusions

The regulation of intestinal drug transporters by the gut microbiota represents a sophisticated multi-tiered interaction between our microbial symbionts and our own physiology. Specific gut microbes and their metabolites (short-chain fatty acids, secondary bile acids, indoles, etc.) can modulate key transport proteins like P-glycoprotein, BCRP, OATPs, and MRPs through well-defined molecular pathways, engaging nuclear receptors (PXR, FXR, AhR, CAR), transcription factors (Nrf2, NF-κB, AP-1), and even direct chemical inhibition of transporter function. These interactions have tangible outcomes on drug pharmacokinetics, influencing how much of an orally administered drug is absorbed, how it is distributed, and the intensity of its effects. They also affect the intestinal barrier’s ability to defend against toxins and maintain immune homeostasis.

Importantly, the microbiota’s influence on drug transporters can be both helpful and harmful. In some cases, it supports the body by speeding up drug elimination, protecting against toxins, and reducing inflammation. In others, it can be problematic, making a drug less effective or even causing toxicity. Therefore, treatment should be personalized: for one patient, strengthening the microbiota might boost transporter activity and lower side effects, while for another, reducing its influence could help improve drug absorption.

**Figure 6:**
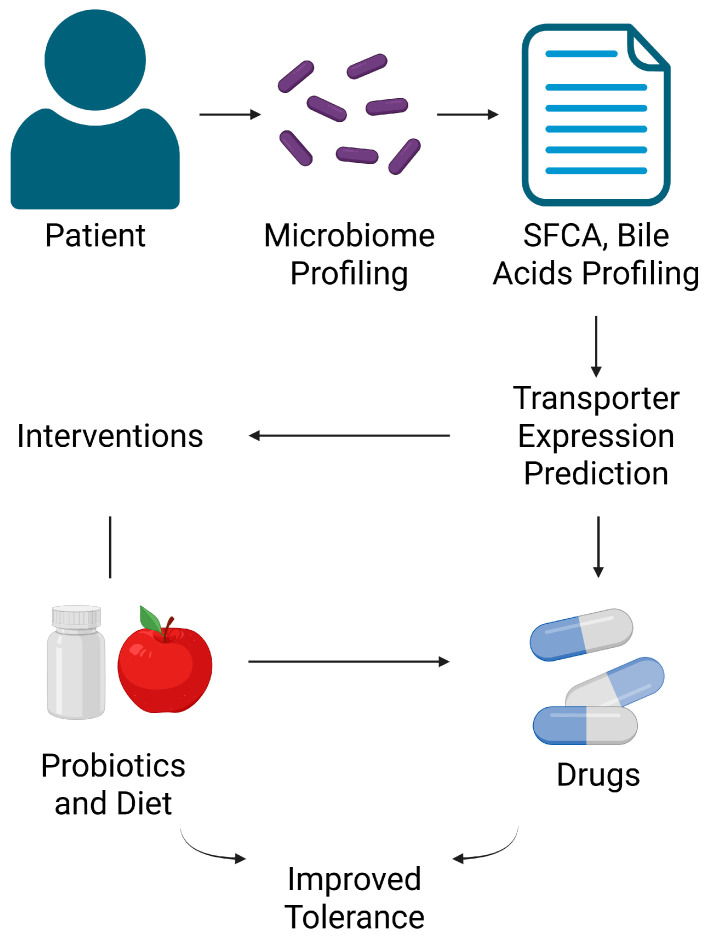
Pharmacomicrobiomics and personalized therapy.

As research progresses, a more integrative pharmacology is emerging, combining microbiomics, genomics, and traditional PK/PD. Clinical management of drugs may soon routinely include microbiome assessments, and interventions like diet or probiotics could become standard adjuncts to modulate drug transport and response. For drug developers and regulatory sciences, considering the microbiome as an active “organ” influencing drug disposition will be important in designing trials and dosage guidelines (Figure 6).

In conclusion, the gut microbiota is a key regulator of intestinal drug transporters through advanced molecular and cellular mechanisms that we are just beginning to fully elucidate. Embracing this knowledge will enhance our ability to predict drug responses, avoid adverse interactions, and personalize therapy. As this field advances, we move closer to a future of precision medicine where not only the patient’s genome but also their microbiome is factored into optimal drug treatment strategies. The old adage “we are what we eat” might be amended to “we absorb what our gut microbes let through,” highlighting the integral role of our microbial partners in pharmacology.

## Figures and Tables

**Table 1:** Major microbiota-derived factors and their known effects on intestinal drug transporters and the underlying host pathways involved.

Microbial Factor (Source)	Host Target Receptor/Pathway	Affected Transporter(s)	Net Effect on Transporter	Reference(s)
Butyrate—short-chain fatty acid produced by *Clostridia* (e.g., *Faecalibacterium*, *Roseburia*) from dietary fiber fermentation.	HDAC inhibition (epigenetic) activates: GPCRs (FFAR2/3, GPR109A) signaling, Nrf2 and other transcription factors.	P-glycoprotein (P-gp, *ABCB1*); possibly MRP2 (*ABCC2*).	Upregulation of transporter expression and function (enhanced efflux activity).	[1]
Secondary bile acids (e.g., deoxycholic acid, lithocholic acid)—produced by microbial 7α-dehydroxylation of primary bile acids (*Clostridium* clusters).	Pregnane X Receptor (PXR) agonism; also FXR and TGR5 activation.	P-gp (*ABCB1*); MRP2 (*ABCC2*); BCRP (*ABCG2*) and others under PXR control.	Upregulation of transporter gene expression (enhanced efflux).	[1]
Tryptophan metabolites (e.g., indole, indole-3-propionic acid)—produced by various commensals from dietary tryptophan.	Aryl hydrocarbon receptor (AhR) activation. AhR/ARNT transcriptional regulation via XREs.	Breast cancer resistance protein (BCRP, *ABCG2*); possibly others with XRE (e.g., Phase I/II enzymes).	Upregulation of transporter expression (enhanced efflux).	[93]
*Lactobacillus* spp. Metabolites (Probiotics)—e.g., *L. acidophilus*, *L. rhamnosus* in gut.	TLR2/agonism or metabolite signaling, MAPK pathway, AP-1 (c-Fos/c-Jun) transcription factor activation.	P-gp (ABCB1).	Upregulation of P-gp expression and function.	[127]
Lipopolysaccharide (LPS)—endotoxin from Gram-negative bacteria (pathobionts or infection).	TLR4 → MyD88 → NF-κB inflammatory signaling; cytokine release (e.g., TNFα, IL-1β).	P-gp (*ABCB1*); BCRP (*ABCG2*); OATP2B1 (*SLCO2B1*); others broadly downregulated by inflammation.	Downregulation of transporter expression; inhibition of transporter function.	[120,121,122,123]
*Eggerthella lenta* metabolites—e.g., secreted isoflavonoid derivatives (from gut Actinobacteria *E. lenta*).	Direct post-translational interaction with P-gp protein (inhibit P-gp ATPase activity).	P-gp (ABCB1).	Functional inhibition of transporter (reduced efflux activity) without changing expression.	[16]
Other microbial factors: H_2_S (sulfide gas from sulfate-reducing bacteria); microbial ROS	Oxidative stress pathways; can modify cysteine residues or signaling (e.g., Keap1/Nrf2 if mild oxidative trigger).	Various (Nrf2 induces MRPs, etc.); direct S-modification can affect protein function.	Generally downregulate transporter if causing inflammation, or mild stress may upregulate via Nrf2.	[115,116,117,118,119]

**Table 2:** Selected Drugs Affected by Microbiota–Transporter Interactions.

Drug (Oral)	Key Transporter(s) Involved	Microbiota/Metabolite Factor	Effect on Drug Pharmacokinetics	Source/Study
Tacrolimus (immunosuppressant)	P-gp (*ABCB1*) efflux in gut; also CYP3A metabolism	Microbiota depletion (antibiotics) → ↑ P-gp expression in small intestine. Commensal metabolites (baseline) keep P-gp lower.	Decreased absorption: Antibiotic-treated or germ-free mice had higher intestinal P-gp and ~50% lower tacrolimus blood AUC vs. controls. In humans, broad antibiotics often reduce tacrolimus levels. P-gp inhibitor reverses this effect.	[17]
Digoxin (cardiac glycoside)	P-gp (*ABCB1*) efflux; minimal metabolism except by gut flora	*Eggerthella lenta* colonization—secretes P-gp ATPase inhibitors; also directly metabolizes digoxin.	Increased absorption rate: *E. lenta* in gnotobiotic mice led to faster and higher early digoxin plasma levels (P-gp inhibited). Total exposure was balanced by microbial degradation of drug. In some patients, *E. lenta* presence correlates with lower digoxin levels (metabolism dominates).	[16,188]
Irinotecan (chemotherapy prodrug)	MRP2 (*ABCC2*) efflux of SN-38 glucuronide into gut; also BCRP efflux of SN-38.	Antibiotic neomycin kills β-glucuronidase bacteria (less SN-38 reactivation); may induce MRP2 via FXR (from accumulated bile acids). Probiotics (*Lactobacillus*) can reduce gut inflammation and maintain transporters.	Decreased toxicity: Neomycin co-treatment in patients reduced incidence of irinotecan-induced diarrhea. Proposed mechanism: more efflux of SN-38 glucuronide (via MRP2) and less bacterial deconjugation. Probiotic trial showed less grade 3 diarrhea. No change in antitumor effect, indicating transport/metabolism alterations were localized.	[194,195,196,197,198]
Metformin (antidiabetic)	OCT1/3 (*SLC22A1/3*) uptake in gut; MDR1/P-gp may affect gut retention; PMAT (SLC29A4).	Microbiota shifts (e.g., ↑ Akkermansia, SCFA producers) with metformin therapy; bile acid changes via microbiota → altered FXR/TGR5 signaling affecting GLP-1 and possibly OCT expression.	Variable effect: Generally, microbiome with more SCFAs (eubiosis) is associated with better metformin response (improved glucose control) and tolerance. Some evidence that microbiota contribute to metformin’s action by increasing GLP-1 (through TGR5) and reducing GI side effects. Transporter-wise, SCFAs may upregulate OCT3 in colon (facilitating metformin uptake into enterocytes for local action).	[191,192,193]

→ indicates “causes” and ↑ indicates “increases”.

## Data Availability

No new data were created or analyzed thus study. Data sharing is not applicable.
